# Finite element analysis of titanium cable tensioning ring fixation in the treatment of distal tibiofibular syndesmosis injury

**DOI:** 10.1097/MD.0000000000048208

**Published:** 2026-04-10

**Authors:** Zuoming Yang, Jianlin Sun, Bin Wang, Pengfei Guan, Xiaoming Zhao

**Affiliations:** a Department of Trauma, The Second Hospital of Tangshan, Tangshan City, Hebei Province, China; b Department of Trauma, Cangzhou Central Hospital, Cangzhou City, Hebei Province, China; c Emergency Department, The Second Hospital of Tangshan, Tangshan City, Hebei Province, China.

**Keywords:** distal tibiofibular syndesmosis injury, endobutton plate, finite element analysis, titanium cable tensioning ring

## Abstract

This study employs the finite element analysis method to compare the biomechanical differences between 2 different internal fixation techniques: the novel surgical approach of using titanium cable tensioning ring for distal tibiofibular syndesmosis (DTS) injuries and the conventional method of utilizing Endobutton plate. Utilizing a CT image of the ankle joint from a healthy adult, models representing a normal ankle joint (Model A), a DTS injury (Model B), a titanium cable tensioning ring (Model C), and an endobutton plate fixation (Model D) were developed through finite element software. The Von Mises stress and fibula displacement relative to the tibia were assessed in each model across 3 loading conditions: standing on 1 leg, ankle internal rotation, and ankle external rotation. Across 3 loading conditions, Model B exhibited the greatest displacement of the fibula compared to Model A, with a maximum rate of increase of 166.3% for anterior and posterior displacement, and 265.7% for internal and external displacement. The fibula displacement rates relative to the tibia in Models C and D were below 20%. Additionally, Model B showed the highest maximum stress on the tibiotalar joint surface, increasing by 42.1%, 41.2%, and 35.0% when standing on 1 foot, pronating inward, and pronating outward, respectively. Conversely, the increase in maximum stress on the tibiotalar joint surface in Models C and D was <5%, resulting in a significant reduction of stress on the tibiotalar joint surface following DTS injury. The results showed that high efficacy in treating DTS injuries was demonstrated by the titanium cable tensioning ring, comparable to the effectiveness of an endobutton plate.

## 1. Introduction

The treatment of Distal tibiofibular syndesmosis (DTS) injuries requires not only the stable fixation of DTS but also the meeting of its micromovement needs, which is a challenging task.^[1–3]^

The traditional treatment technique for DTS injury is cortical bone screw fixation, which is controversial in terms of the exact number and method used. Some clinical practitioners have replaced it with elastic fixation represented by tabbed plates because of the difficulty in meeting the micromobility requirements of DTS, the presence of various complications, such as nail breakage, and the need to undergo several surgeries.

Tabbed plates achieve elastic fixation of the DTS via fiber wires, which can combine stable fixation and allow micromovement of the DTS. The procedure is more biomechanical and does not have the problems of nail breakage and individual removal. In a clinical study,^[4]^ patients with DTS injuries treated with tabbed plates showed better outcomes than those treated with conventional cortical bone screw fixation in terms of postoperative imaging evaluation and long-term prognosis.

However, tabbed steel plates are more expensive, cause greater damage to the bone, are relatively cumbersome to operate intraoperatively, exhibit difficulty while adjusting the elasticity, and may become loose.^[5]^ Therefore, some researchers have developed an elastic fixation method, such as titanium cable loop ligation, for treating DTS injuries. Mechanical experiments on cadaveric specimens^[6]^ showed that the titanium cable ligature fixation device can provide sufficient tensile strength for treating DTS injuries, and its biomechanical efficacy is higher than that of cortical bone screws. In clinical application,^[7]^ 1-year postoperative X-ray images of patients with DTS injuries treated with titanium cable isotonic rings showed that the titanium cable fixation device was positioned in the correct location in all patients, with no loosening or fracture. However, studies on the comparison between the efficacy of titanium cable annular fixation devices and tabbed steel plates are limited; therefore, these 2 elastic fixation modalities were selected for the experiment.

Due to the limitations of traditional studies on autopsy and the scarcity of resources, many studies are using electronic information technology. Finite element analysis techniques allow quantitative data to be obtained simultaneously by applying different treatment protocols and different values of stress to the same model, thus minimizing confounding factors, which is not possible with conventional experiments.

In this study, we evaluated and analyzed the biomechanical characteristics of 2 internal fixation modalities, including a titanium cable isotonic ring and tabbed steel plate, for treating DTS injuries using the finite element analysis method. In order to fill the gap in this field, this study might provide a novel theoretical basis for the treatment of clinical DTS injury.

## 2. Materials and methods

### 2.1. Data collection and modeling

A CT image of an ankle joint of a healthy adult volunteer was collected, and the geometrical model of the bone was constructed by removing mania using the Mimics 21 software (Materialise, Inc.) and developing boundary thresholds for each bone. Further patching and optimization were performed using the Geomagic 2021 software (Geomagic Inc.) to remove the non-characteristic protrusions and indentations on the model surface, and then, smooth surfaces were used to fit the triangular facets of the model surface. Finally, a surface model with continuity was generated. This study has been approved by the Technical Committee and Ethics Committee of The Second Hospital of Tangshan, with ethics approval number of The Second Hospital of Tangshan, (2024) No. (22). Volunteers have provided sufficient risk notification and signed informed consent forms.

The models of each part were imported into the SolidWorks software (Dassault) and assembled according to the origin fit. Next, cartilage was added to each articular surface to build a model of a normal ankle joint consisting of the tibia, fibula, talus, Achilles, and cartilage (denoted as Model A). The titanium cable isotonic ring and tabbed steel plate were reconstructed using the SolidWorks software. In Model A, 2.5 cm above the articular surface of the distal tibia, a bone channel (2.0 mm) was drilled from the center of the lateral cortex of the fibula to the tibia at an angle of 30° from posterior to anterior. Then, a second channel was drilled along the posterior margin of the ankle and parallel to the first one to the tibia, and then, a titanium cable (1.3 mm in diameter) was threaded through it in circles to establish a preliminary model of titanium isotachymetry ring fixation. In the other Model A, 2.5 cm above the articular surface of the distal tibia, a foramen (3.2 mm in diameter) was made at the center of the lateral cortex of the fibula by penetrating 4 layers of the cortex at an angle of 30° from the posterior exterior to the anterior interior. Then, plates were placed in the lateral fibula and the medial tibia, respectively, to establish a preliminary model of fixation with a collapsed steel plate.

### 2.2. Material properties

The component structures in the model were considered to be uniformly distributed, isotropic, and linearly elastic. The properties of the component materials were assigned values based on published studies (Table 1).^[8]^

**Table 1:** Properties of individual component materials.

	Young modulus (MPa)	Poisson ratio
Bone	7300	0.3
Gristle	12	0.42
Titanium	110,000	0.3

In this experiment, based on the properties of each ligament, different numbers of stretch-only springs were used to simulate the ligament attachments. In total, 31 springs were set up by referring to the anatomical parameters of the relevant ligament attachment points,^[9,10]^ and wider ligaments were simulated with multiple springs. We established the ligamentous structures of AITFL, PITFL, TL, anterior talofibular ligament, posterior talofibular ligament, Achilles fibular ligament, anterior tibial talofibular ligament, posterior tibial talofibular ligament, tibial-heel ligament, and interosseous membrane in this experiment. Considering the different mechanical characteristics of each ligament, the corresponding stiffness values were assigned to each ligament based on the experimental data from published studies.^[11]^ The interosseous membrane of the lower leg was represented by 5 springs, each with a stiffness value of 400 N/mm; all other ligaments for which no data were found in the literature or for which the data varied considerably were considered to have stiffness values between 70 and 90 N/mm^12^. The fiber wires connecting the medial and lateral tabbed plates were simulated by 2 springs with a stiffness value of 1000 N/mm. In the normal ankle model, the preliminary model of isotonic ring fixation with a titanium cable, and the preliminary model of plate fixation with tabs, the spring modules representing the AITFL, PITFL, TL, and interosseous membrane close to the tibial talonavicular joint in the inner part (8 cm) were compressed to obtain the DTS injury model (denoted as Model B), the model of isotonic ring fixation with titanium cable (denoted as Model C), and the model of plate fixation with tabs (denoted as Model D), respectively.

### 2.3. Boundary conditions and loads

Each cartilage was set as a bound joint with its corresponding bone, and the interaction between bones was set as surface contact to suppress crossover. Interference was detected to exclude bone overlap caused by small changes in the model surface fitting process. Based on the lubricating property of the surface of the articular cartilage, the friction coefficient between cartilages in the joint was set to 0.01, and the effect of gravity was ignored in this experiment.

By constraining all the nodes below the heel bone and talus, the center point of the upper end of the tibia and fibula was fixed in the X-axis and Y-axis directions, and the remaining parts were set to be freely movable. When humans stand on 1 foot, the knee joint bears about 85.6% of the body mass.^[12]^ All models simulated the situation when standing on 1 foot, and the body mass of the volunteer in this experiment was 70 kg. Thus, a load of 600 N was applied to the upper end of the tibia, extending down the longitudinal axis of the tibia. Then, a clockwise and counterclockwise torsional moment of 2.7 N-m was applied to simulate the internal and external rotation motion of the lower limb with the longitudinal axis of the tibia as the center of rotation while keeping the constraint and the vertical load constant.

### 2.4. Mesh division

The 10-node tetrahedral cells were used to mesh each part of the model using a high-quality mesh generation tool. Models A and B had 429,178 nodes and 293,972 cells; Model C had 456,585 nodes and 313,204 cells; Model D had 433,162 nodes and 296,179 cells.

### 2.5. Observational indicators

Iterative calculations were performed using the simulation static analysis module, focusing on comparing the model tibial talonavicular joint surface Von Mises stress and fibula displacement relative to the tibia.

## 3. Results

### 3.1. Validation of the model

This experiment tested the maximum displacements and the location of the maximum displacement distribution of a normal ankle joint model under 3 different loads, including 1-legged stance, ankle internal rotation, and ankle external rotation. By comparing the maximum displacements and the location of the maximum displacement distribution of the normal ankle joint model under 3 different loads with published literature, the effectiveness of the model is verified.

Liu et al^[13]^ analyzed the biomechanics of the ankle joint after cortical bone screw fixation using the 3-dimensional finite element method. The normal ankle joint model they established had maximum model displacements of 1.31, 3.84, and 2.70 mm in 1-legged stance, ankle internal rotation, and ankle external rotation, respectively. The maximum displacements of the normal ankle joint model established in this experiment in 1-legged standing position, ankle internal rotation, and ankle external rotation, as well as, the location of the maximum displacement distribution matched the findings of other studies, which confirmed that the model established in this experiment was valid. The 4 groups of models established in this experiment were completely parallel, which enabled a more accurate assessment of the differences in the observed indicators between the models.

### 3.2. Comparison of displacement and distribution

**Figure 1.**
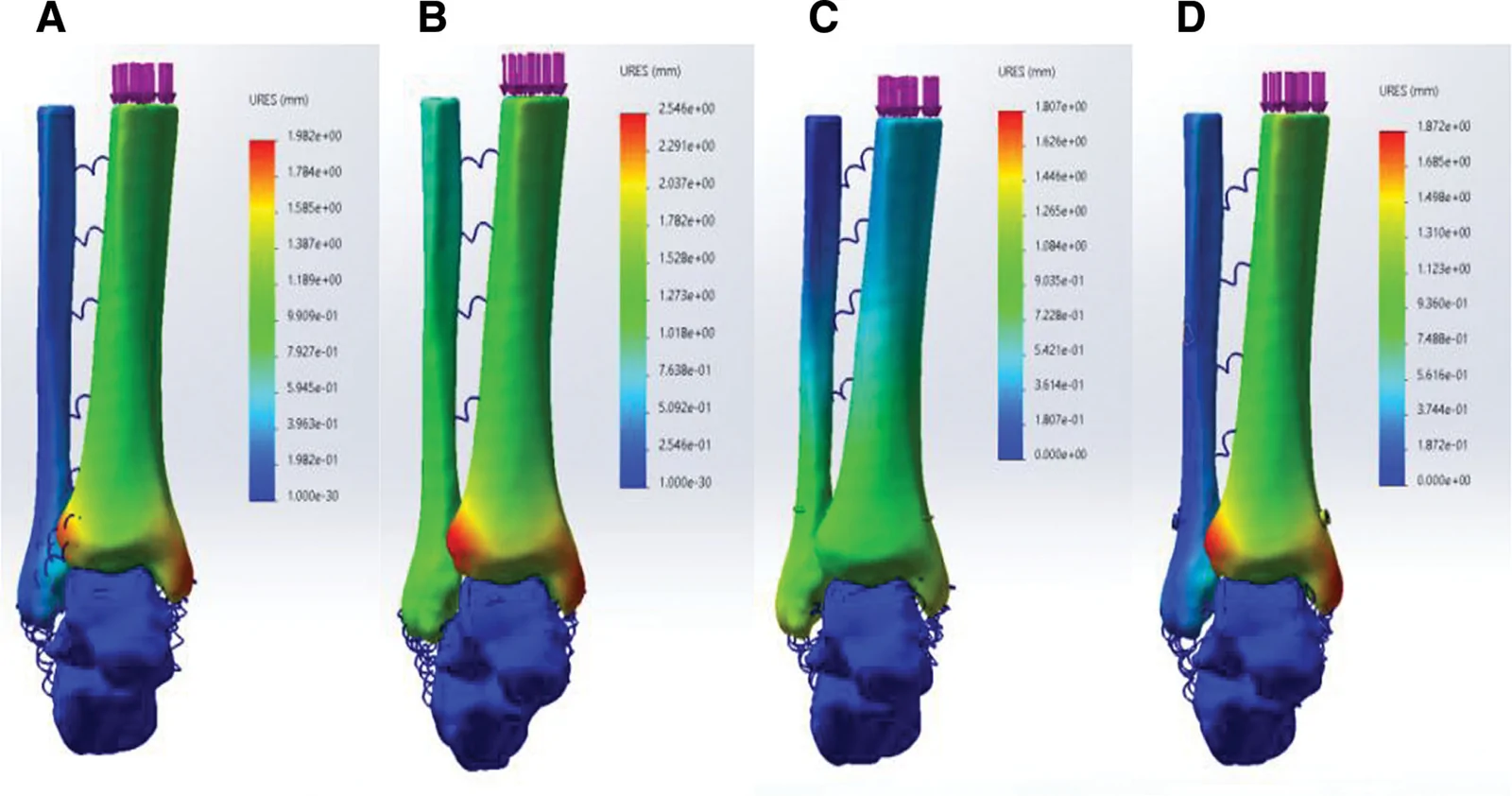
Displacement distribution of the 4 models in the 1-legged standing position. (A) Normal ankle; (B) DTS injury; (C) titanium cable isotonic ring fixation; (D) plate fixation with tabs. DTS = distal tibiofibular syndesmosis.

**Figure 2.**
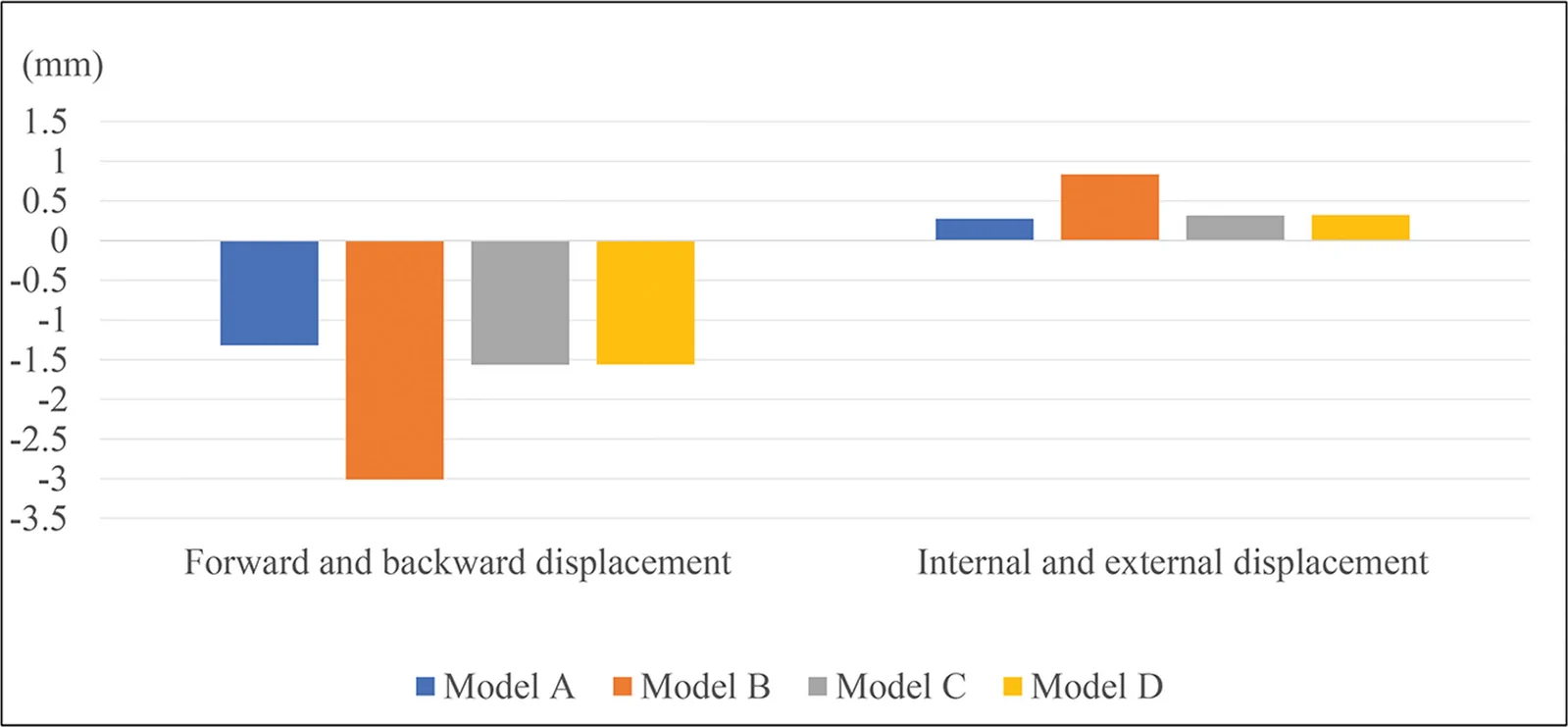
Displacement of the fibula relative to the tibia in a 2-cm cross-section of the tibial talocalcaneal joint surface when standing on 1 leg. Backward and outward displacements are positive, while forward and inward displacements are negative.

The anteroposterior displacement of the fibula relative to the tibia in a 2-cm cross-section of the tibial talonavicular articular surface in Models B, C, and D was different from that of Model A by 128.0%, 18.6%, and 18.1% for the 1-legged standing position, and the internal and external displacements differed from that of Model A by 203.6%, 14.9%, and 17.5%, respectively (Tables 2 and 3, Figs. 1 and 2).

**Table 2:** Anteroposterior and posterior displacement of the fibula relative to the tibia in a 2-cm cross-section on the tibial talar articular surface in the 4 model groups (in mm).

	Model A	Model B	The difference ratio of Model A	Model C	The difference ratio of Model A	Model D	The difference ratio of Model A
Unipedal stand	1.320	3.009	128.0%	1.565	18.6%	1.559	18.1%
Internal rotation of the ankle joint	1.154	3.073	166.3%	1.303	12.9%	1.342	16.3%
External rotation of the ankle joint	1.021	2.101	105.8%	1.117	9.4%	1.097	7.4%

**Table 3:** Internal and external displacement of the fibula relative to the tibia in a 2-cm cross-section on the articular surface of the tibial talonavicular in the 4 model groups (in mm).

	Model A	Model B	The difference ratio of Model A	Model C	The difference ratio of Model A	Model D	The difference ratio of Model A
Unipedal stand	0.275	0.835	203.6%	0.316	14.9%	0.323	17.5%
Internal rotation of the ankle joint	0.347	0.575	265.7%	0.294	15.3%	0.281	19.0%
External rotation of the ankle joint	0.973	2.031	108.7%	1.057	8.6%	1.069	9.9%

**Figure 3.**
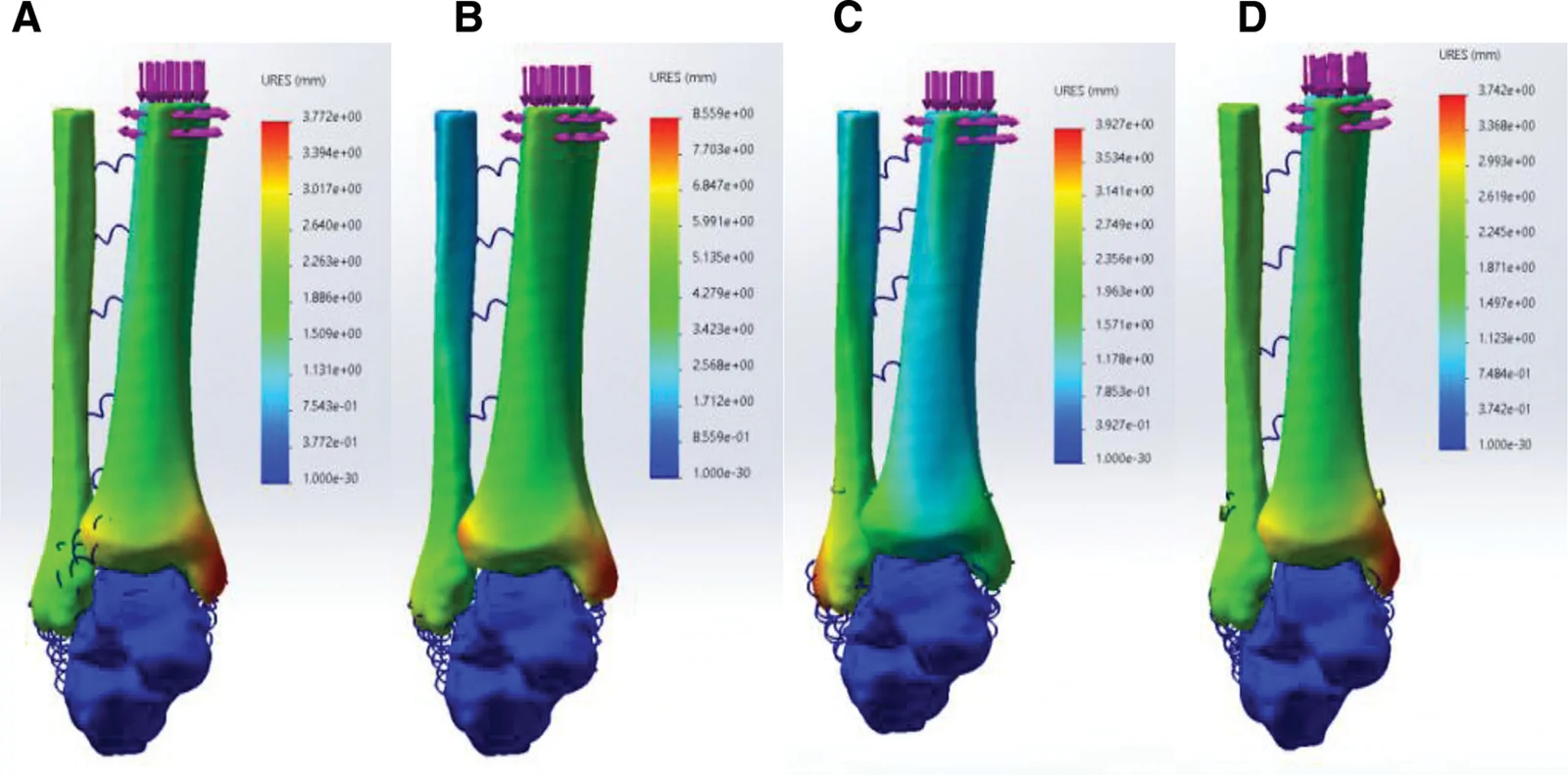
Displacement distribution of the 4 models during internal rotation. (A) Normal ankle; (B) DTS injury; (C) titanium cable isotonic ring fixation; (D) plate fixation with tabs. DTS = distal tibiofibular syndesmosis.

**Figure 4.**
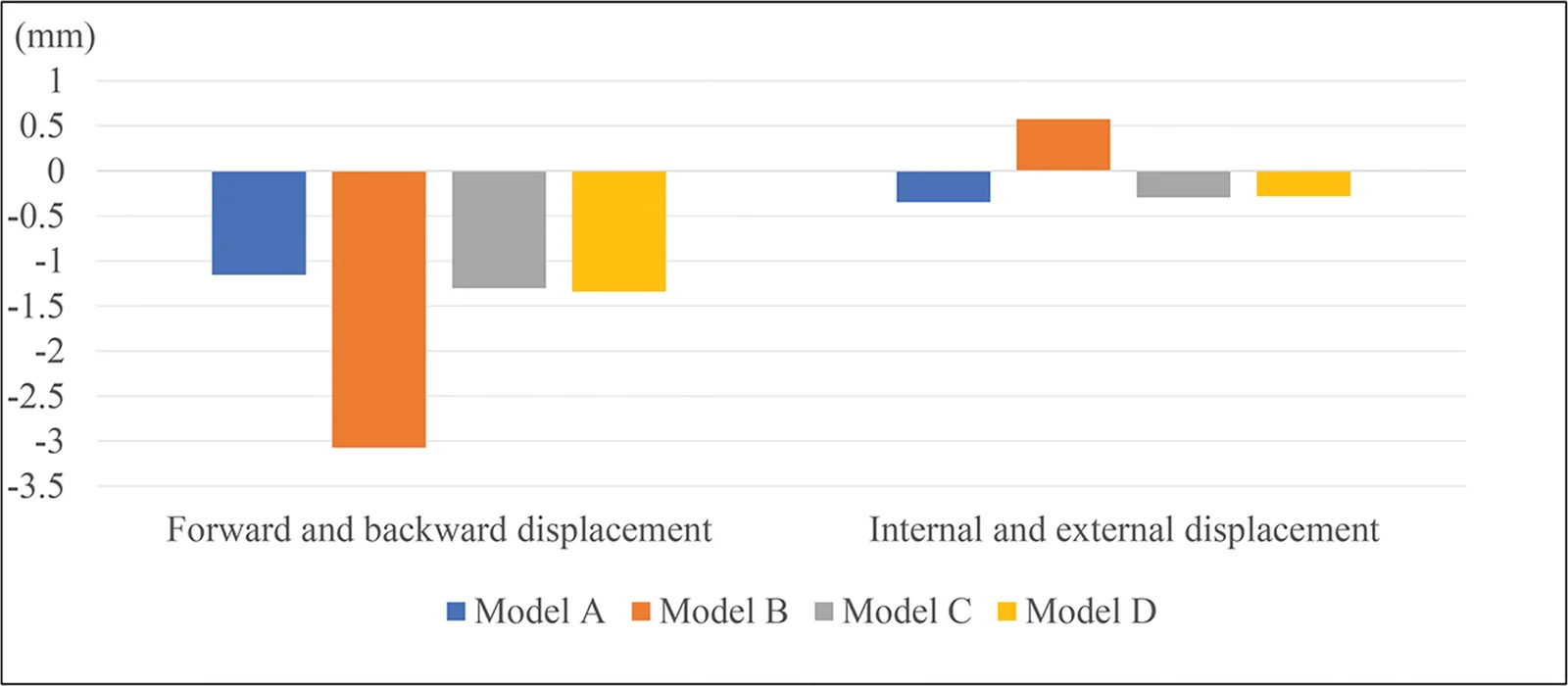
Displacement of the fibula relative to the tibia in a 2-cm cross-section on the articular surface of the tibial talonavicular joint during ankle internal rotation. Backward and outward displacements are positive, while forward and inward displacements are negative.

During the internal rotation of the ankle, the anteroposterior displacement of the fibula relative to the tibia in a 2-cm cross-section on the articular surface of the tibial talonavicular joint in Models B, C, and D differed from Model A by 166.3%, 12.9%, and 16.3%, and the internal and external displacements differed from Model A by 265.7%, 15.3%, and 19.0%, respectively (Table 2 and 3, Figs. 3 and 4).

**Figure 5.**
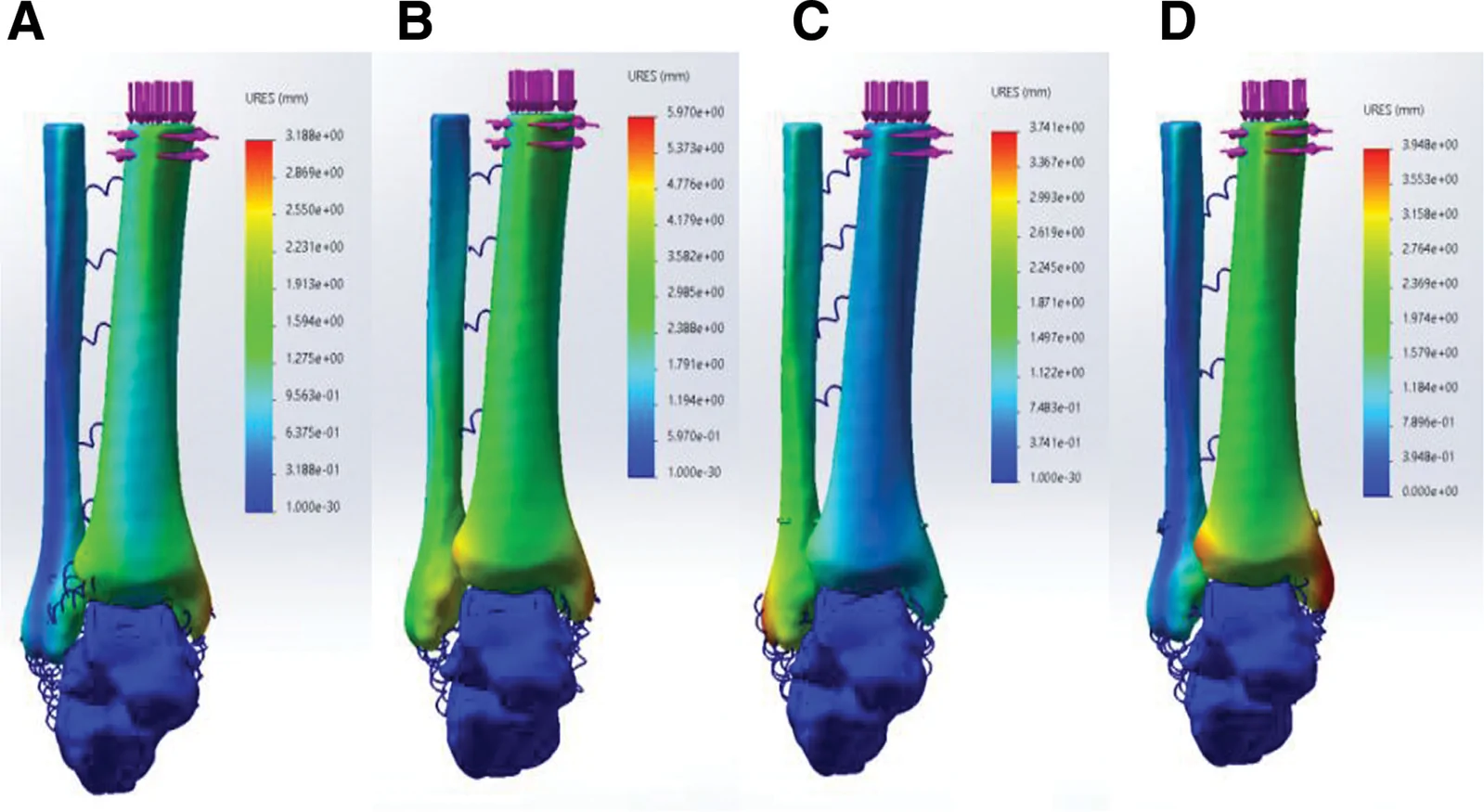
Displacement distribution of the 4 models during external rotation. (A) Normal ankle; (B) DTS injury; (C) titanium cable isotonic ring fixation; (D) plate fixation with tabs. DTS = distal tibiofibular syndesmosis.

**Figure 6.**
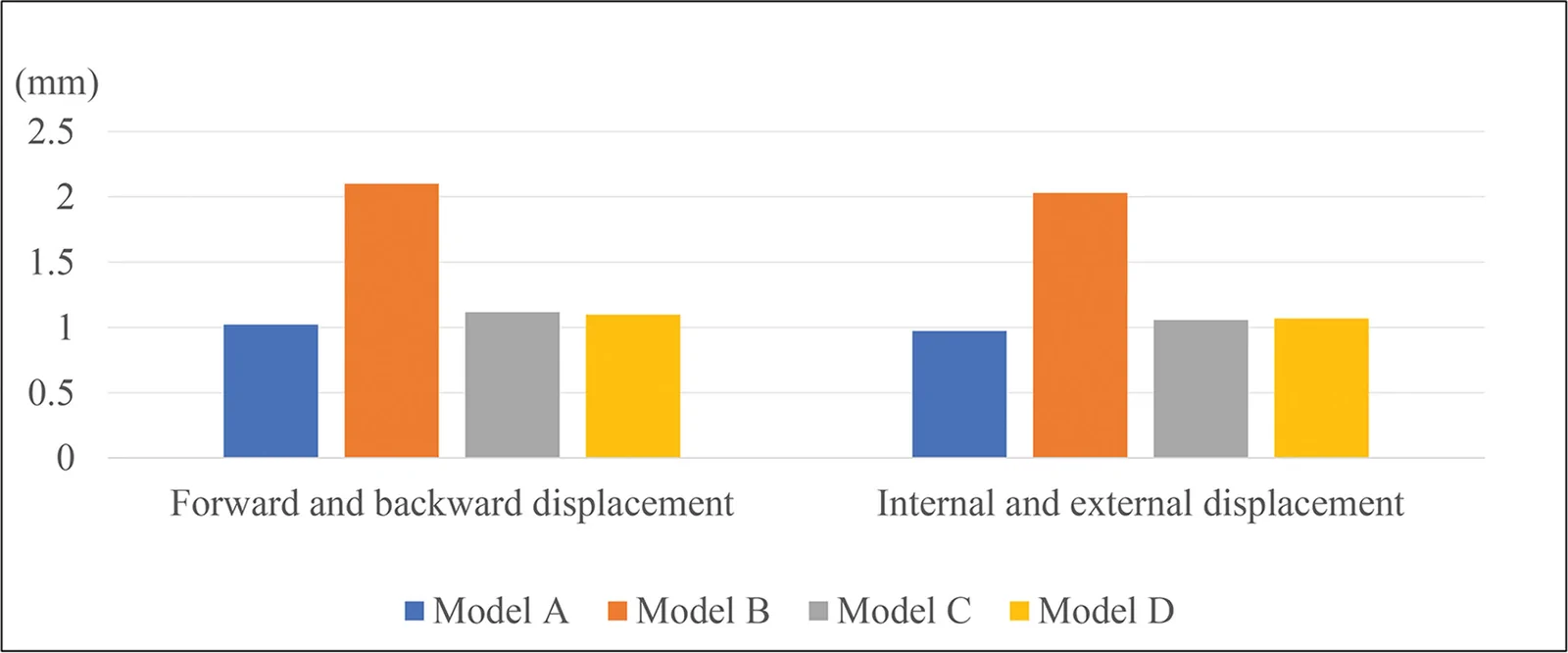
Displacement of the fibula relative to the tibia in a 2-cm cross-section on the articular surface of the tibial talonavicular joint during ankle external rotation. Backward and outward displacements are positive, while forward and inward displacements are negative.

During external rotation of the ankle, the anteroposterior displacement of the fibula relative to the tibia in a 2-cm cross-section on the articular surface of the tibial talonavicular joint in Models B, C, and D differed from Model A by 105.8%, 9.4%, and 7.4%, and the internal and external displacements differed from Model A by 108.7%, 8.6%, and 9.9%, respectively (Tables 2 and 3, Figs. 5 and 6).

### 3.3. Comparison of stress distribution

**Figure 7.**
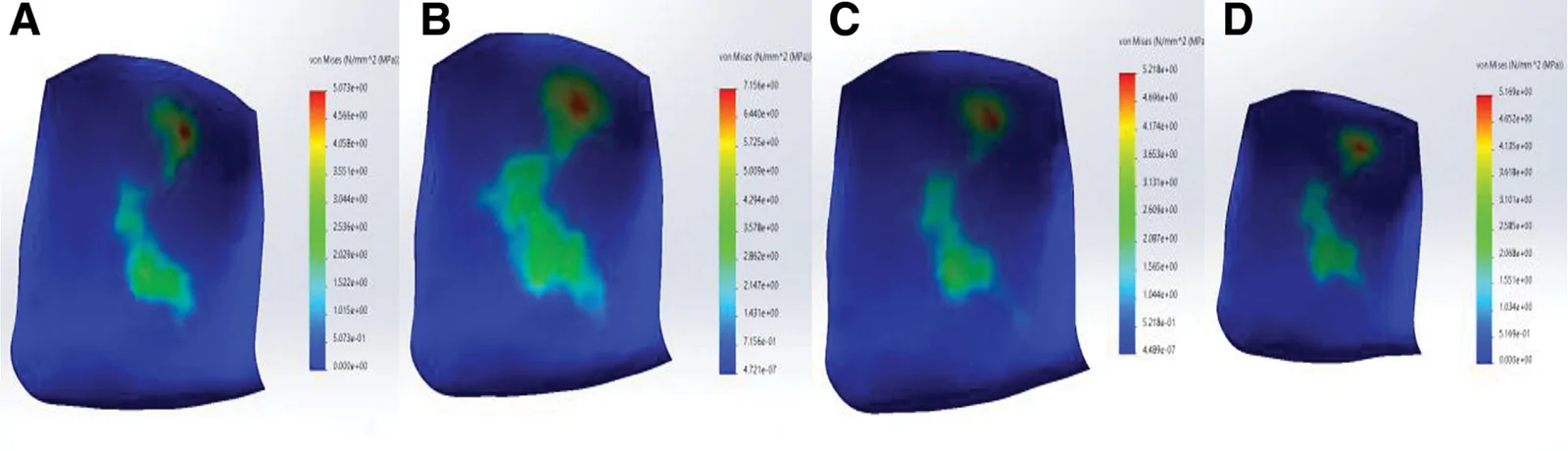
Tibial talonavicular joint surface stress for the 4 models related to the 1-legged stance. (A) Normal ankle; (B) DTS injury; (C) titanium cable isotonic ring fixation; (D) plate fixation with tabs. DTS = distal tibiofibular syndesmosis.

**Figure 8.**
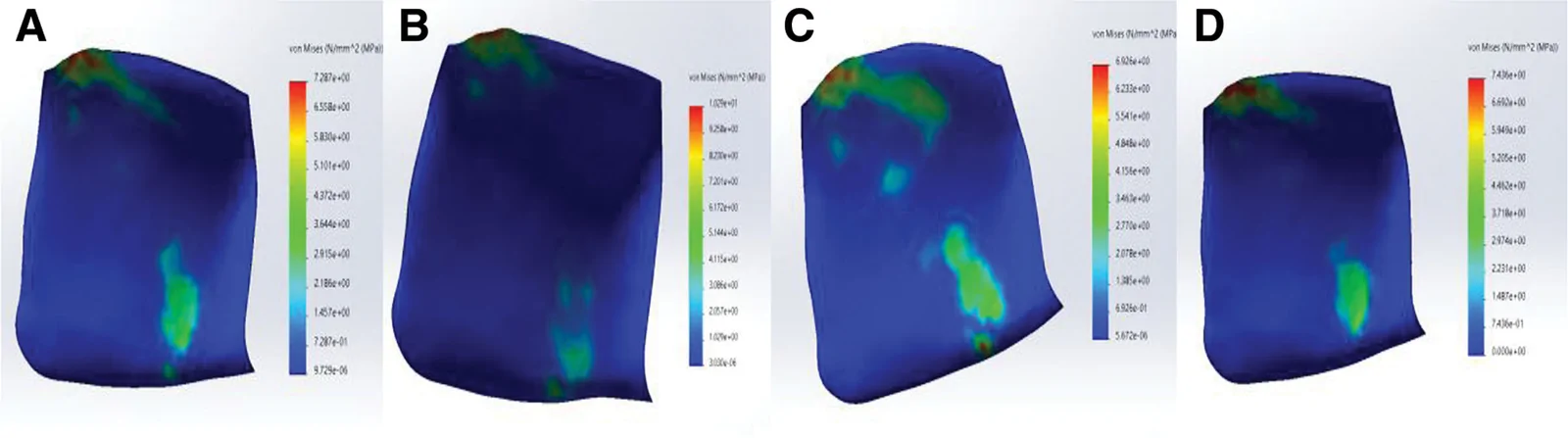
Tibial talonavicular joint surface stress for the 4 models related to internal rotation. (A) Normal ankle; (B) DTS injury; (C) titanium cable isotonic ring fixation; (D) plate fixation with tabs. DTS = distal tibiofibular syndesmosis.

**Figure 9.**
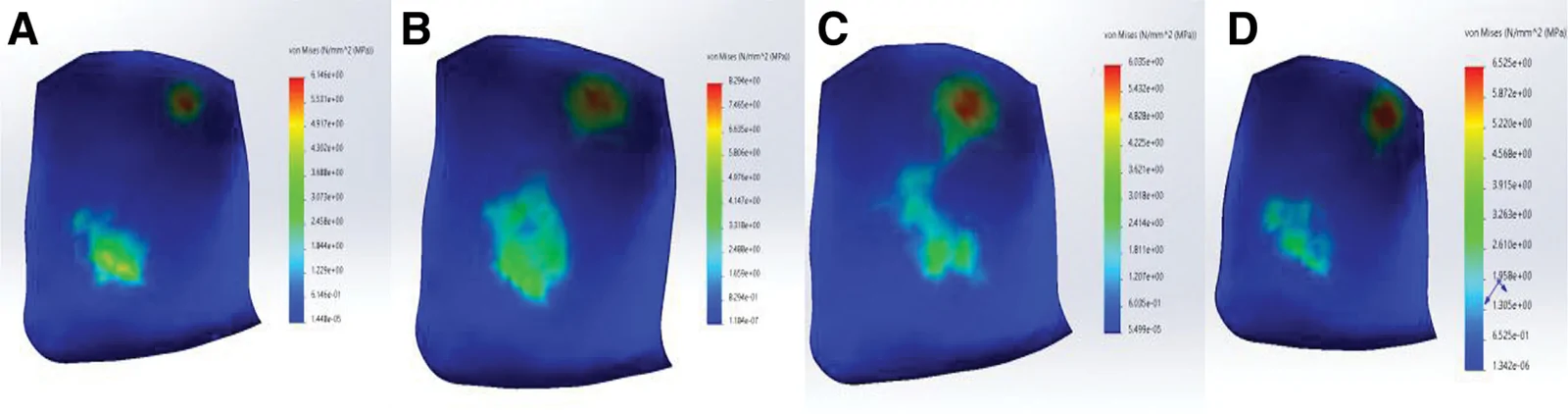
Tibial talonavicular joint surface stress for the 4 models related to external rotation. (A) Normal ankle; (B) DTS injury; (C) titanium cable isotonic ring fixation; (D) plate fixation with tabs. DTS = distal tibiofibular syndesmosis.

**Figure 10.**
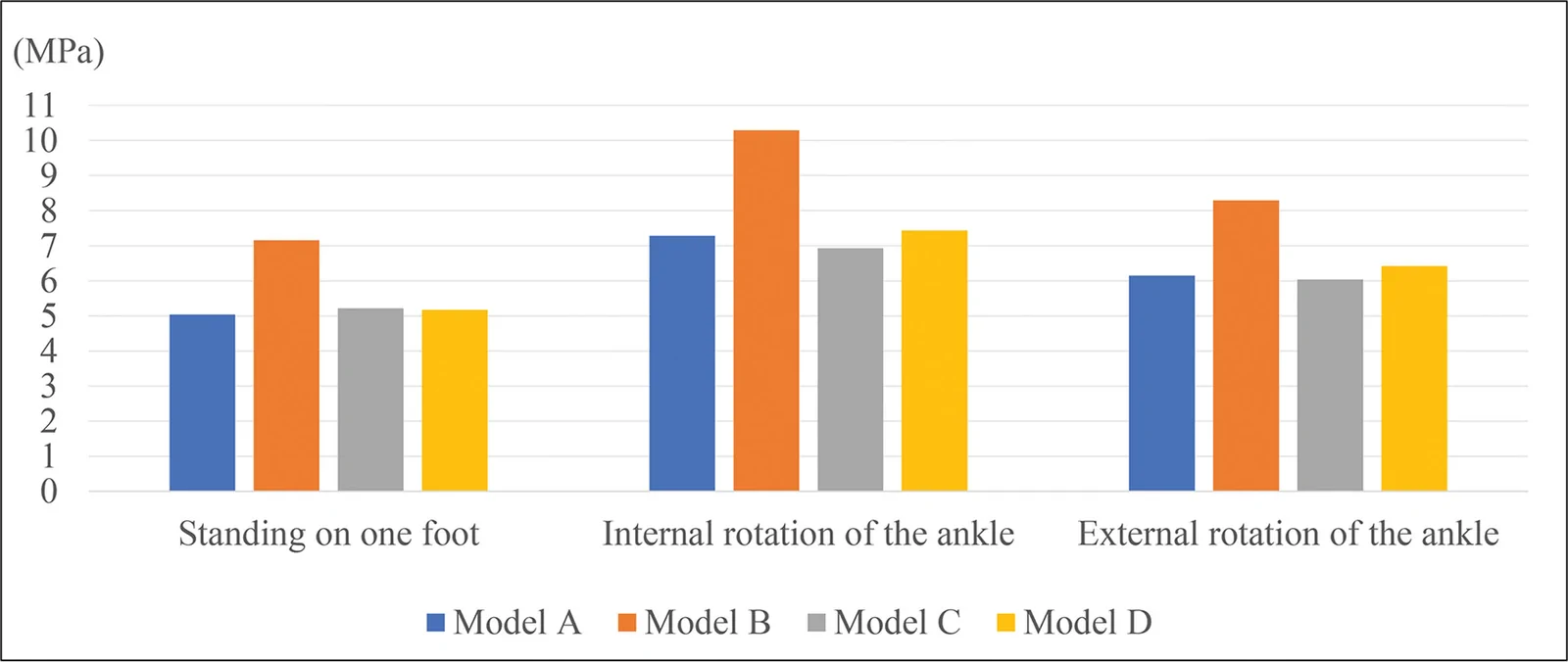
The maximum stress in the tibial talonavicular joint surface for each model under 3 loads.

The difference ratios of the maximum stress in the articular surfaces of the tibial talonavicular joints were 42.1%, 3.6%, and 2.6% for Models B, C, and D, respectively, compared to Model A. In the single-leg stance, the ratios were 41.2%, 4.9%, and 2.0%, respectively, for internal rotation of the ankle joint, and 35.0%, 1.8%, and 4.5%, respectively, for external rotation of the ankle joint (Table 4, Figs. 7–10).

**Table 4:** Maximum stress in the tibial talonavicular joint surfaces of the 4 model groups (MPa).

	Model A	Model B	The difference ratio of Model A	Model C	The difference ratio of Model A	Model D	The difference ratio of Model A
Unipedal stand	5.037	7.156	42.1%	5.218	3.6%	5.169	2.6%
Internal rotation of the ankle joint	7.287	10.291	41.2%	6.926	4.9%	7.436	2.0%
External rotation of the ankle joint	6.146	8.294	35.0%	6.035	1.8%	6.425	4.5%

## 4. Discussion

Simple DTS ligament injuries caused by ankle sprains can be classified into 3 grades according to the different degrees of injury,^[14]^ and DTS ligament grade III injuries require surgical treatment. This experimental injury model simulated the DTS ligament grade III injury. Clinically, DTS injuries are usually combined with ankle fractures, to simplify the model, and the fracture is considered to be strongly fixed so that the model only simulates the DTS injury and the 2 treatment modalities.

During the normal physiological movement of the ankle joint, the external ankle can adjust its relative position as the ankle joint moves, and it can move slightly within a range of 2 to 5° of rotation^[15]^ and 1 to 2 mm of anterior-posterior translation,^[16]^ which in turn maintains the stability of the ankle joint. The movement of the outer ankle is more pronounced during internal and external rotation; thus, the 3 most representative loads of single-leg neutral, ankle internal rotation, and ankle external rotation were selected for analysis.^[13,17]^

The results of the finite element analysis showed that the relative displacement between the fibula and tibia increased significantly under the 3 loads in Model B, and the stress concentration in the articular surface of the tibial talonavicular joint indicated that the complete injury of the DTS greatly affected the stability of the ankle joint and increased the risk of the articular surface injury, as reported in another study,^[18]^ and surgical treatment was required.

The displacement of the fibula relative to the tibia under the 3 loads in Models C and D was significantly lower than that in Model B and was similar to that in Model A. This indicated that the titanium cable isotonic ring and the tabbed steel plate maintained the stability of the DTS and enabled micromovement within the physiological range, which met the needs of clinical treatment.

Under the 3 loading conditions, the maximum stress in the tibial talonavicular joint surface in Models C and D was significantly lower than that in Model B and was similar to that in Model A, with a difference ratio of <5%. This finding indicated that the titanium cable isotonic ring and the tabbed steel plate significantly decreased the stress in the tibial talonavicular joint surface after the DTS injury, restoring it to a level close to that of the normal ankle joint, and decreasing the risk of further injury to the articular surface in the long term.

This study had some limitations. First, the model in this study was obtained from CT images of only 1 patient, and the material properties assigned were obtained from previous studies. Thus, the experimental results have some differences. Additionally, the activity of the ankle joint is more complex under real conditions, and ligaments, muscles, joint capsules, and other soft tissues should be involved. However, the mechanical characteristics of other materials cannot be determined easily. We aim to collect more data through cadaveric biomechanical studies, which might improve the ability to simulate the 3-dimensional finite element analysis.

To summarize, DTS injury leads to relative displacement between the fibula and the tibia and a significant increase in the stress on the tibial talar joint surface. The titanium cable isotonic ring and the tabbed steel plate can effectively immobilize DTS injuries, retaining their micromovement properties while enabling stable immobilization of the strength of the DTS and significantly reducing the stress on the tibial talar joint surface, thus decreasing the risk of articular surface injuries.

At present, there are many studies on the clinical efficacy of Endobutton plate in literature, but most of them are compared with traditional metal screws. In addition, reports on titanium cable tensioning ring are limited to a very small number of clinical and autopsy studies, and the quantity is very limited. The comparative study between these 2 methods is even more blank. The biomechanical analysis results of 2 treatment methods in this study are consistent with relevant literature reports.

The results of the experiment only reflected the early postoperative effect and could not provide information on the long-term internal fixation failure. Additionally, the observation was limited to the stress and displacement of the ankle joint under 3 kinds of motion, including neutral, internal rotation, and external rotation. In the future, composite simulation studies may be performed on the dorsiflexion and plantarflexion of the ankle joint and other motions.

## Author contributions

**Conceptualization:** Zuoming Yang, Jianlin Sun, Bin Wang, Pengfei Guan, Xiaoming Zhao.

**Data curation:** Zuoming Yang, Jianlin Sun.

**Formal analysis:** Jianlin Sun.

**Funding acquisition:** Jianlin Sun.

**Investigation:** Jianlin Sun.

**Methodology:** Jianlin Sun.

**Project administration:** Jianlin Sun.

**Resources:** Jianlin Sun.

**Software:** Zuoming Yang.

**Supervision:** Jianlin Sun.

**Validation:** Zuoming Yang, Jianlin Sun.

**Visualization:** Zuoming Yang, Jianlin Sun.

**Writing –original draft:** Zuoming Yang.

**Writing – review & editing:** Zuoming Yang, Jianlin Sun, Bin Wang, Pengfei Guan, Xiaoming Zhao.
